# Effects of Dried Blueberry Pomace and Pineapple Pomace on Growth Performance and Meat Quality of Broiler Chickens

**DOI:** 10.3390/ani13132198

**Published:** 2023-07-05

**Authors:** Yaodong Hu, Shi Tang, Wei Zhao, Silu Wang, Caiyun Sun, Binlong Chen, Yuxing Zhu

**Affiliations:** College of Animal Science, Xichang University, Xichang 615000, China

**Keywords:** blueberry pomace, pineapple pomace, growth performance, meat quality, broiler chicken

## Abstract

**Simple Summary:**

Pomace, which is produced by fresh fruit after juice pressing, is rich in protein, crude fiber, vitamins, polyphenols, and so on. Making full use of pomace as feed in the breeding industry can reduce the need for many feed ingredients. Therefore, this study evaluated the effects of adding 3% blueberry pomace and pineapple pomace to the diet of broiler chickens on their growth performance and the quality of their meat. The results show that adding dried pomace to broiler chickens’ diets did not reduce growth performance or meat quality. This study provides a new approach and a reference for the development and utilization of broiler feed ingredients.

**Abstract:**

The purpose of this study was to investigate the effects of dried blueberry pomace (BP) and pineapple pomace (PP) on the growth performance and meat quality of broiler chickens. A total of 240 1-day-old Ross 308 broiler chickens were randomly divided into 3 groups, with 10 replicates per treatment group and 8 birds per replicate (4 males and 4 females). The three groups were the control (CON) group, the 3% BP group, and 3% PP group. The entire trial period lasted 42 days. The results show that the average daily feed intake, average daily gain, and feed-to-gain ratio of the BP group and the PP group were not significantly different from those in the CON group (*p* > 0.05). Adding BP to the diet significantly reduced the proportion of liver and giblets (*p* < 0.05). Adding PP to the diet significantly reduced the proportion of liver, while the proportion of gizzard significantly increased (*p* < 0.05). The pH^24h^ and L* of breast muscles were significantly lower in the PP group than in the CON group (*p* < 0.05). The water-holding capacity of the leg muscles in the BP group and the PP group was significantly lower than that in the CON group (*p* < 0.05). The crude protein content of breast muscle and the ether extract content of leg muscle in the BP group were significantly lower than those in the CON group (*p* < 0.05). In conclusion, the addition of 3% BP and PP to broiler chickens’ diets had no adverse effects on growth performance or meat quality.

## 1. Introduction

Chicken, as a source of high-quality animal protein, is widely popular, and the consumer demand for chicken is still growing around the world [1]. To meet people’s increasing consumption demand, some studies have shown that the global feed supply will need to exceed 7 billion tons [2]. As the main cost of poultry farming (more than 70%), feed cost affects breeding efficiency and breeding enthusiasm [3,4]. These costs can be effectively controlled through the reasonable formulation of rations from a nutritional point of view [5]. The development and utilization of new feed ingredients and the search for other, cheaper feed ingredients to replace bulk feed ingredients are some effective ways to control costs.

Pomace is a by-product of fruit juice obtained through the squeezing or crushing of fruit. Although pomace contains a core, peel, stem, and stalk, more than 70% of it can be effectively used [6,7]. In addition, most of the nutrients remaining in pomace are equivalent to those in the fruit, which has a high feed utilization value. The composition of nutrients in pomace varies depending on the type of fruit. Previous studies have shown that pomace is rich in vitamins, minerals, polyphenols, and dietary fiber, with possibly higher contents than in fruit [8,9]. These residual substances can provide essential nutrients for poultry production. In addition, some special fruit residues have been shown to be rich in protein and oil, which provide certain nutritional value for poultry [8,9]. Studies have shown that the world produces 500 million tons of pomace from juice production. Because the output of pomace is too large, in order to save on the cost of pomace treatment, fruit juice production plants generally sell pomace at a low price. Although some pomace is used for food processing, effective substance extraction, etc., there is still no large-scale development or utilization of pomace in the world [10,11]. Unutilized pomace can be an environmental pollutant due to its rapid deterioration. Therefore, using some pomace in poultry feed can effectively solve the problem of environmental pollution and reduce feed cost.

There have been some studies on the feasibility of adding pomace to poultry feed in the early stage. Olive pomace contains high concentrations of phenols, lipids, and organics, and a wide range of nutrients [12]. Preliminary studies have shown that adding 100 g/kg and 150 g/kg olive pomace to the diet of broiler chickens did not negatively affect their performance [13,14]. Adding 1% olive pomace to the diet of layers did not increase egg production [15]. Low levels of olive pomace (4%) could effectively improve the growth performance of broiler chickens [16]. Apple pomace contains large amounts of carbohydrates, crude fiber, trace elements, polyphenols, and pectin [17]. A study found that adding apple pomace to broiler chickens’ diets could increase body weight for six weeks, but this effect was not significant compared with that seen in the control group [18]. Therefore, these studies suggest that fruit pomace could be effectively utilized in poultry diets, but further evaluation of the nutritional effects on different types of fruit pomace was needed.

Blueberries contain a large number of polyphenols, such as anthocyanins and flavanols, which have been proven to be effective in fighting oxidative stress and inflammation and lowering cholesterol. It is a health food that is loved by people. Similarly, the blueberry pomace (BP) that remains (20% of fruit) after squeezing the juice also contains large amounts of carbohydrates, proteins, lipids, minerals, and polyphenols [19]. Pineapple is a tropical fruit and the most valuable fruit of the bromeliad family. Despite its sweetness, pineapple is low in calories and packed with vitamins, antioxidants, and enzymes, which help boost the immune system and digestion [20]. Pineapples are processed to produce pineapple pomace (PP, 25–35%), which includes the peel (30%), pomace (50%), stone (7%), and crown (13%) [21]. Both kinds of the abovementioned pomace, olive pomace and apple pomace, have the same nutrients, including polyphenols, vitamins, minerals, proteins, etc., and have a certain utilization value. However, whether they are safe feed ingredients in poultry feed needs further research. Moreover, there is still a lack of research on using the two types of pomaces in cage broiler chickens’ diets. Therefore, the main aim of this study was to investigate the effect of replacing 3% of broiler chickens’ diet with BP and PP on the growth performance and meat quality of broiler chickens.

## 2. Materials and Methods

### 2.1. Animals, Diets, and Experimental Design

A total of 240 one-day-old healthy and mixed-sex Ross 308 broiler chickens with a similar initial body weight (BW) were purchased from Liangshan Meigu County Yanying Agricultural Technology Co., Ltd. (Liangshan prefecture, China). They were randomly divided into 3 treatment groups; each treatment group had 10 replicates, and each replicate had 8 chickens (4 males and 4 females). The experimental diets were the basal diet group (CON), BP group, and PP group. Dried BP and dried PP were used to replace 3% corn in the basal diet in the BP and PP groups, respectively. The composition and nutrient level of the basal diet are shown in Table 1. BP and PP were purchased from local fruit juice producers. The specific nutritional components were measured in our laboratory after drying (Table 2). All birds were housed in steel cages (140 cm × 60 cm × 50 cm). All cages were equipped with one feeder and four drinkers. The whole experiment lasted 42 days in total. All birds had free access to food and water. The light lasted 24 h from 1 to 3 days and 23 h of light and 1 h of darkness during days 4–42. The temperature of the chicken house was maintained at 33 °C for the first week and gradually dropped by 3 °C every week. Humidity in the house was maintained at 65%.

### 2.2. Sample Collection

The BW of each bird was measured on days 0, 21 and 42. The feed intake (FI) for each cage was recorded every day. After the experiment, 10 birds (1 bird per replicate; sex ratio was 1:1) were selected from each treatment group, sacrificed via neck dislocation, and dissected. The giblets, breast muscles, leg muscles, leg bones, abdominal fat, liver, gizzard, and heart were separately weighed. The dressing percentage and the proportion of each tissue to the carcass were calculated from the obtained weight data. All of the obtained samples were stored at 4 °C for further determination

### 2.3. pH, Meat Color, Drip Loss, and Water-Holding Capacity Analysis

The pH of the meat was measured at 45 min and 24 h with a tester model PH2108 (Spectronics, New York, NY, USA). The meat color of the predissection carcass, the breast muscles, and leg muscles after dissection was measured with a meat color measuring instrument OPTO-STAR (Matthaus, Berlin, Germany). We weighed 80 g of the breast muscles or leg muscles to airtight containers, which we then refrigerated at 4 °C for 24 h to measure the weight loss as drip loss. We weighed 300 mg of breast muscles or leg muscles on the center of filter paper and sandwiched it between two glass plates. Then, we pressed it with two kilograms of pressure for 5 min. After the extrusion, the proportion of the stained area and the sample area were measured separately. The water holding capacity (WHC) was calculated from the percentage of the difference between the stained area and the sample area.

### 2.4. Nutrient Analysis 

Breast muscles and leg muscles were ground and pulverized for nutritional analysis. Pomace samples were used to analyze the amount of dry matter (DM), crude protein (CP), ether extract (EE), crude fiber (CF), energy, and minerals (calcium, magnesium, and phosphorus). Muscle tissue samples were used to analyze the contents of DM, CP, EE, and crude ash. The crude protein in the meat was determined via the drying method (AOAC, method 950.46). The EE was determined after the meat was mixed with part of the sand, dried, and extracted with ether (AOAC, method 960.39). The CP content in meat was obtained by multiplying the nitrogen content in the meat by 6.25 (AOAC, method 970.42). The amount of DM in the pomace was determined by drying at 135 °C for 2 h (AOAC, method 930.15). The crude protein concentration of the diet and pomace samples was determined via the combustion method (AOAC, method 990.03). The calcium, magnesium, and phosphorus contents of the diets and pomace samples were analyzed via inductively coupled plasma spectroscopy (AOAC, method 985.01). The pomace fiber concentration was determined by the AOAC method 2002.04 [22]. The EE in the pomace was determined via Soxhlet extraction. The energy in the pomace samples was measured using a fully automatic nitrogen-oxygen energy meter (BYLRY-3000 W, Beijing Hongda Boyu Technology Co., Ltd., Beijing, China). After acid treatment, the diets were measured using a fully automatic amino acid analyzer (HITACHI -LA8080, Hitachi, Ltd., Tokyo, Japan).

### 2.5. Statistical Analyses

Statistical analyses were carried out using SAS software version 9.4 P. The experimental unit for growth performance was the pen (cages), while an individual bird selected from each replicate was the experimental unit for the other measured indices. The results were analyzed with one-way ANOVA. Diet was a fixed effect, and weight and sex were random effects. Significant differences between experimental groups were determined with Duncan’s test. *p* < 0.05 meant a significant difference. Values between *p* > 0.05 and *p* < 0.10 were considered a significant trend. *p* > 0.10 meant no significant difference.

## 3. Results

### 3.1. Growth Performance

There was no significant difference in the growth performance of broiler chickens with the addition of dried BP or PP (Table 3; *p* > 0.05). However, there were upward trends in final body weight (FBW; day 42) and average daily weight gain (ADG; day 1–42).

### 3.2. Carcass Traits

There was a significant decrease in the giblet weight of broiler chickens with the addition of dried BP (Table 4; *p* < 0.05). Both BP and PP supplementation very significantly reduced the percentage of liver in broiler chickens (*p* < 0.05). The addition of PP significantly increased the percentage of gizzards of broiler chickens (*p* < 0.05).

### 3.3. Meat Quality and Meat Color

The addition of dried BP and PP did not significantly affect the pH^45min^ (Table 5; *p* > 0.05). However, the pH^24h^ of breast muscles decreased with dried PP supplementation (*p* < 0.05). There was an obvious decrease in the drip loss of leg muscles with dried BP supplementation (*p* < 0.05). Both dried BP and PP supplementation decreased the WHC of leg muscles (*p* < 0.05). The addition of dried BP and PP did not affect the drip loss or WHC of the breast muscles (*p* > 0.05). The addition of dried PP decreased the L* of the breast muscles (Table 6; *p* < 0.05). There were no significant differences in the carcass or leg muscles with the addition of dried BP or PP (*p* > 0.05). Both the a* and b* of carcass, breast muscles, and leg muscles showed no significant change when dried BP and PP were added to the diet of broiler chickens (*p* > 0.05).

### 3.4. Nutritional Level in the Muscles

The crude ash and EE of the leg muscles significantly decreased with dried BP supplementation (Table 7; *p* < 0.05), while those of breast muscles were not significantly different with the addition of both BP and PP (*p* > 0.05). Dried BP and PP supplementation did not affect (*p* > 0.05) the DM or CP of the breast and leg muscles versus those of the CON broiler chickens. Adding PP effectively increased the content of CP in the breast muscle compared with that in the PP group (*p* > 0.05).

## 4. Discussion

Growth performance directly reflects the growth of broiler chickens, and it is an indicator with which feed managers are concerned. The results of this study showed that replacing 3% of dried BP or dried PP in broiler chickens’ diets had no significant effect on the growth performance of broiler chickens. In addition, we found that the addition of 3% dried BP and dried PP increased BW on day 42 and ADG during days 1 to 42, but not significantly. A study showed that the FI of the 2% blueberry extract treatment group was significantly higher than that of the control group throughout the test period, and the addition of blueberry extract could effectively improve the feed conversion efficiency of broiler chickens from day 22 to day 42 [23]. Moreover, the reason for the difference in the above results may be that the blueberry extract contained higher levels of nutrients and antioxidant substances such as polyphenols than BP and PP. Although this study did not measure the content of polyphenols in the pomace, many studies have shown that PP and BP contain polyphenols. The polyphenolic substances in blueberries can improve the digestibility of dietary amino acids, proteins, and energy [24,25,26]. In addition, polyphenols protect the body from oxidative damage through free radical scavenging activity, reducing lipid peroxidation, and activating antioxidant pathways [27,28]. In this study, we found that the BP group tended have higher BW and ADG. Based on the above results, it was speculated that increasing the proportion of BP (increasing the content of active ingredients) would likely promote the growth performance of broiler chickens. However, this conjecture needs to be further verified, because with increasing amounts of BP, the content of CF and other substances correspondingly increased, which may have a certain negative impact on the growth performance of broiler chickens. So far, no research has been conducted on adding PP to the diets of broiler chickens. However, this study showed that adding 3% dried PP to the diets of broiler chickens had no significant effect on growth performance compared with that of the CON group. Although the energy value of both pomaces (3.56 kcal/g and 2.68 kcal/g) was lower than that of corn (3.93 kcal/g), the addition of 3% pomace did not reduce growth performance. Therefore, it is feasible to replace 3% corn with PP or BP in broiler chickens’ diets. 

Our study found that the proportion of giblets and liver to carcass was significantly lower in the BP group; in the PP group, the proportion of liver was significantly lower but the proportion of gizzard was significantly higher than in the CON group. However, the slaughter rate was not affected by treatment. Most previous studies have shown that polyphenolic compounds do not affect broiler chickens’ carcass yield or characteristics [24,25,29]. However, broiler chickens fed 2% blueberry extract had a significantly higher proportion of gizzard [23]. The possible reason for the change in liver ratio was that the polyphenols altered nutrient metabolism, especially lipid metabolism. The main reasons for the differences between the studies may be the differences in the source, type, content, and composition of polyphenols.

In addition, this study investigated the effect of adding pomace to broiler chickens’ diets on meat quality. We found that pH^24h^ was significantly lower in the PP group than in the CON group. This may have been due to the rich content of ascorbic acid in PP. We also observed that the leg muscles’ drip loss in the BP group and leg muscles’ WHC of the BP and PP groups were significantly lower than those of the CON group. This result showed that the addition of pomace can increase the water absorption of broiler chickens’ leg muscles.

Meat color is a visual indicator that people use to choose meat products. Different customers judge the freshness and tenderness of meat by their preference for different meat colors. Previous research showed that consumers prefer whiter meat [30]. Another study showed that the lower the L* value and the higher the a* and b* value, the more popular the meat color. Pomace is also a source of natural dyes such as carotenoids, flavonoids and anthocyanins [31,32]. In this study, it was observed that the L* value of the breast muscles in the PP group was significantly lower than that in the CON group. 

Finally, we also determined the nutritional composition of the muscles. Nutritional value is also an important indicator for meat quality [33]. We found that the BP group had significantly lower crude ash and EE contents in the leg muscles than the CON group. Polyphenolic compounds had antioxidant properties. Broiler chickens absorb polyphenols and transported them to different tissues of the body [25]. Studies have shown that polyphenols can scavenge free radicals in breast and leg muscle tissues, increase liver glutathione levels [27,28], and reduce lipid peroxidation [25]. The above studies have shown that polyphenols and other oxidants can change the metabolism of nutrients and cause differences in the nutritional level of meat. 

## 5. Conclusions 

The addition of 3% dried BP and dried PP to broiler chickens’ diets had no adverse effect on growth performance or meat quality. Therefore, it is feasible to add 3% BP or PP to broiler chickens’ diets.

## Figures and Tables

**Table 1:** Basal diet composition and nutrient level in different phases.

Items	Day 1–21	Day 22–42
Ingredients		
Corn	58.00	63.00
Soybean meal (43% CP)	31.60	27.00
Corn gluten meal (63% CP)	3.00	2.00
Soybean oil	3.00	4.00
Limestone	1.23	1.30
Dicalcium phosphate	2.00	1.60
L-lysine	0.32	0.30
DL-methionine	0.15	0.10
Sodium chloride	0.30	0.30
Vitamin premix ^1^	0.20	0.20
Mineral premix ^2^	0.20	0.20
Total	100.00	100.00
Calculated nutrient levels		
Apparent metabolizable energy, MJ/kg	12.78	12.63
Crude protein	21.55	19.02
Calcium	1.06	0.96
Available phosphorus	0.47	0.38
Lysine	1.21	1.13
Methionine	0.51	0.46
Methionine + cystine	0.91	0.76
Analyzed nutrient levels		
Crude protein	21.34	19.22
Calcium	0.97	0.99
Total phosphorus	0.79	0.64
Methionine	0.46	0.45
Lysine	1.12	1.08

^1^ Premix provided per kilogram of diet: vitamin A, 10,000 IU; vitamin D3, 3000 IU; vitamin E, 30 IU; menadione, 1.3 mg; thiamin, 2.2 mg; riboflavin, 8 mg; nicotinamide, 40 mg; choline chloride, 600 mg; calcium pantothenate, 10 mg; pyridoxine HCl, 4 mg; biotin, 0.04 mg; folic acid, 1 mg; vitamin B12, 0.013 mg. ^2^ Premix provided per kilogram of diet: Fe, 80 mg; Cu, 8.0 mg; Mn, 110 mg; Zn, 60 mg; I, 1.1 mg; Se, 0.3 mg.

**Table 2:** Nutrient level of dried blueberry pomace and pineapple pomace.

Item, g/kg	BP	PP
Dry matter	954.8	924.5
Crude protein	82.3	55.6
Crude fat	58.2	11.2
Crude fiber	118.3	183.8
Energy value, kcal/g	3.56	2.68
Calcium	1.43	1.96
Magnesium	0.26	0.98
Phosphorus	3.65	4.21

Note: BP, dried blueberry pomace; PP, dried pineapple pomace.

**Table 3:** Effects of dried blueberry pomace and pineapple pomace on growth performance of broiler chickens.

Item	CON	BP	PP	SEM	*p*-Value
Initial body weight, g	41.44	41.40	41.51	0.182	0.967
Day 21 body weight, g	760.2	761.2	764.2	6.335	0.898
Day 42 body weight, g	2359.5	2384.6	2381.6	8.555	0.095
Average daily feed intake, g					
Day 1–21	48.94	49.20	48.91	0.205	0.554
Day 22–42	144.7	147.1	151.0	2.219	0.148
Day 1–42	96.20	99.80	98.90	1.361	0.170
Average daily weight gain, g					
Day 1–21	34.23	34.27	34.41	0.298	0.898
Day 22–42	76.16	77.30	77.02	0.466	0.212
Day 1–42	55.19	55.79	55.72	0.204	0.096
Feed-to-gain ratio					
Day 1–21	1.431	1.437	1.422	0.013	0.727
Day 22–42	1.902	1.903	1.962	0.036	0.405
Day 1–42	1.743	1.790	1.776	0.028	0.490

Note: CON, basal diet; BP, 3% dried blueberry pomace diet; PP, 3% dried pineapple pomace diet; SEM, standard error of means.

**Table 4:** Effects of dried blueberry pomace and pineapple pomace on carcass traits of broiler chickens. (*n* = 10).

Item, %	CON	BP	PP	SEM	*p*-Value
Dressing percentage with giblets	75.67	75.51	75.96	0.174	0.198
Dressing percentage without giblets	71.41	71.42	71.75	0.146	0.193
Breast muscle	23.25	23.45	23.38	0.132	0.561
Leg muscle	20.48	20.39	20.45	0.085	0.746
Leg bones	5.587	5.619	5.565	0.046	0.670
Abdominal fat	2.195	2.147	2.162	0.026	0.410
Giblets	5.540 ^a^	5.335 ^b^	5.387 ^ab^	0.057	0.046
Liver	3.338 ^a^	3.041 ^c^	3.168 ^b^	0.021	<0.001
Gizzard	1.814 ^b^	1.874 ^b^	1.908 ^a^	0.017	0.003
Heart	0.744	0.743	0.785	0.015	0.081

Note: CON, basal diet; BP, 3% dried blueberry pomace diet; PP, 3% dried pineapple pomace diet; SEM, standard error of means. ^a,b,c^ Means in the same row with different superscripts differ significantly (*p* < 0.05).

**Table 5:** Effects of dried blueberry pomace and pineapple pomace on meat quality of broiler chickens (*n* = 10).

Item	CON	BP	PP	SEM	*p*-Value
pH^45min^					
Breast muscles	6.451	6.458	6.443	0.012	0.670
Leg muscles	6.668	6.703	6.679	0.029	0.693
pH^24h^					
Breast muscles	5.939 ^a^	5.906 ^ab^	5.877 ^b^	0.015	0.025
Leg muscles	6.468	6.505	6.533	0.023	0.152
Drip loss, %					
Breast muscles	0.747	0.752	0.740	0.009	0.650
Leg muscles	0.440 ^a^	0.395 ^b^	0.432 ^a^	0.009	0.002
Water holding capacity, %					
Breast muscles	18.48	18.56	18.34	0.225	0.785
Leg muscles	16.55 ^a^	15.87 ^b^	15.84 ^b^	0.182	0.016

Note: CON, basal diet; BP, 3% dried blueberry pomace diet; PP, 3% dried pineapple pomace diet; SEM, standard error of means. ^a,b^ Means in the same row with different superscripts differ significantly (*p* < 0.05).

**Table 6:** Effects of dried blueberry pomace and pineapple pomace on meat color of broiler chickens. (*n* = 10).

Item	CON	BP	PP	SEM	*p*-Value
L*					
Carcass	65.22	64.42	65.10	0.274	0.102
Breast muscles	56.31 ^a^	55.59 ^ab^	55.27 ^b^	0.271	0.034
Leg muscles	57.99	57.30	57.03	0.339	0.138
a*					
Carcass	10.35	10.42	10.39	0.095	0.873
Breast muscles	12.04	12.05	11.78	0.166	0.439
Leg muscles	45.75	45.81	45.45	0.225	0.488
b*					
Carcass	13.95	13.71	14.25	0.183	0.133
Breast muscles	9.249	8.930	9.218	0.192	0.444
Leg muscles	9.557	9.560	9.290	0.105	0.131

Note: CON, basal diet; BP, 3% dried blueberry pomace diet; PP, 3% dried pineapple pomace diet; L*, lightness; a*, redness; b*, yellowness. SEM, standard error of means. ^a,b^ Means in the same row with different superscripts differ significantly (*p* < 0.05).

**Table 7:** Effects of dried blueberry pomace and pineapple pomace on the nutritional level in the muscles of broiler chickens.

Item, %	CON	BP	PP	SEM	*p*-Value
Dry matter					
Breast muscles	24.82	25.06	24.32	0.286	0.195
Leg muscles	24.70	24.86	24.24	0.291	0.310
Crude ash					
Breast muscles	1.146	1.141	1.135	0.006	0.478
Leg muscles	1.098 ^a^	1.010 ^b^	1.085 ^a^	0.018	0.003
Crude protein					
Breast muscles	24.50 ^ab^	24.30 ^b^	25.01 ^a^	0.178	0.025
Leg muscles	20.41	20.23	20.69	0.176	0.194
Ether extract					
Breast muscles	1.111	1.131	1.213	0.030	0.059
Leg muscles	4.458 ^a^	4.265 ^b^	4.346 ^ab^	0.044	0.015

Note: CON, basal diet; BP, 3% dried blueberry pomace diet; PP, 3% dried pineapple pomace diet; SEM, standard error of means. ^a,b^ Means in the same row with different superscripts differ significantly (*p* < 0.05).

## Data Availability

Data will be made available upon reasonable request.
